# Towards a comprehensive psychosocial profile of the family caregiver of people with dementia

**DOI:** 10.1186/s41155-025-00342-5

**Published:** 2025-03-20

**Authors:** Sara Jiménez García-Tizón, Ana Belén Navarro Prados, María Belén Bueno Martínez

**Affiliations:** Departamento de Psicología Evolutiva y de la Educación, Facultad de Psicología, Universidad de Salamanca, Salamanca, Spain

**Keywords:** Care, Dementia, Family caregiver, Profile, Psychoeducational intervention

## Abstract

**Background:**

The profile of caregivers focuses on socio-demographic variables (age, gender, kinship, and educational level). However, buffer variables (e.g., dysfunctional thoughts) and indicators of the impacts of caregiving (e.g., stress) are often overlooked.

**Objective:**

The study aims to offer a comprehensive view of the profile of the family caregiver of people with dementia by considering aspects contemplated in theoretical explanatory models of care.

**Methods:**

It was based on a cross-sectional design. Socio-demographic and care-related characteristics and variables of the consequences of caregiving and related buffers were evaluated for 40 family caregivers of people with dementia. The Short form of Social Problem-Solving Inventory-Revised (SPSI-R-25), the Leisure Time Satisfaction Survey (LTS), the Revised Scale for Caregiving Self-Efficacy (RSCSE), the *Escala de Habilidades Sociales* (EHS), the Psychosocial Support Questionnaire (PSQ), the *Cuestionario de Pensamientos Disfuncionales* (CPD), the Caregiver Burden Interview (CBI), the Center for Epidemiologic Studies-Depression Scale (CES-D), the Perceived Stress Scale (PSS) and the World Health Organization Quality of Life Assessment–AGE (WHOQOL-AGE) were utilized. Descriptions of the variables and contrast tests (chi-square tests, *t*-tests, and two-factor ANOVAs) were made and used to compare the differences according to gender and kinship.

**Results:**

Family caregivers are women, typically around 60 years old, wives or daughters, married, with primary/secondary education, who spend long hours caring with little support. They presented a low level of social and problem-solving skills, received little social support, had low self-efficacy and quality of life, highly dysfunctional thoughts, overload, depressive symptomatology, and stress. Significant differences were found according to kinship in educational level, employment status, hours per day dedicated to care time, and contemplating placing the person in a nursing home. Significant differences by kinship were also found in self-efficacy for responding to disruptive behaviors, perceived social support, and stress. Spouses have lower levels of this type of self-efficacy and have higher levels of stress but perceive greater social support. Males have higher levels of dysfunctional thoughts.

**Conclusion:**

This study facilitates the identification of the specific needs of caregivers, to provide them with individualized interventions. Spouses and offspring present different needs and therefore interventions should be different.

**Supplementary Information:**

The online version contains supplementary material available at 10.1186/s41155-025-00342-5.

## Introduction

The growth of the elderly population has resulted in a concomitant increase in the prevalence of chronic and degenerative diseases, such as dementia. Dementia is a syndrome defined by an impairment of cognitive function, which is the ability to process thoughts. Impairment of cognitive function affects memory, thinking, orientation, comprehension, calculation, learning, language, and judgment. Alzheimer’s disease is the most prevalent form of dementia, accounting for 60–70% of cases. Other forms include vascular dementia, dementia with Lewy bodies, and a group of diseases that contribute to frontotemporal dementia. The development of dementia may also be precipitated by a stroke or in the context of certain infections (e.g., Human Immunodeficiency Virus (HIV)), due to the harmful use of alcohol, repetitive physical injuries to the brain, or nutritional deficiencies (The World Health Organization, WHO, 2023).

Advanced age and dependency, or the need for assistance, are closely related issues that often compel elderly people to require the attention or care of others (*Instituto de Mayores y Servicios Sociales*, IMSERSO, 2015). Globally, the number of people with dementia is approximately 55 million, with 10 million new cases being diagnosed each year. In 2030, this number is expected to increase to 82 million, and 152 million in 2050 (WHO, 2023). Consequently, the situation of caring for a person with dementia is becoming ever more frequent among societies.

In Spain, family caregivers are the primary care providers. Likewise, this type of assistance constitutes the fundamental basis of social care policies for the elderly in developed countries that advocate for the concept of “aging at home” (Frías-Osuna et al., 2018; Petrini et al., 2019). However, this responsibility is typically shouldered by a single family member who assumes the role of the main caregiver. Currently, this role is often assumed by women between the ages of 50–60 years old. These women are typically daughters of dementia sufferers, housewives, married, and with a basic level of primary education (Navarro-Abal et al., 2019; Pérez et al., 2020).

Despite their great work, family caregivers often receive little recognition or support. During the process of becoming a caregiver, they undergo various phases of adaptation. This new situation generates several changes in one’s personal, family, and social life, and frequently physical, psychological, and socio-family alterations tend to appear (Cascella & García-Orellán, 2020; González-de Paz et al., 2016).

The different theoretical models associated with care posit that caring for a person with dementia involves a state of chronic stress for the caregiver. Most of them have agreed that there are variables (buffer variables, such as dysfunctional thoughts and social or problem-solving skills…) that modulate the consequences (e.g., stress, depressive symptomatology, overload, or quality of life) that care can have on him or her.

In particular, the Multidimensional Model of Stress (Schulz et al., 2000) and the Multidimensional Stress Model Applied to Care (Pearlin et al., 1990) (models on which this study is based) have posited that the effects on the physical, emotional, and social functioning of the caregivers depend on how they assess their situation, that is, on the stressors. An assessment characterized by threat and perceived lack of coping capacities will lead to negative consequences; while an assessment as perceived benign or with the perception of having sufficient coping capacities will, on the contrary, lead to positive consequences. Each assessment will be influenced by the background and current context in which the caregiver operates; nevertheless, it is essential to acknowledge the critical influence of buffer factors or variables. Analyzing the buffer variables is essential for ensuring that the caregiving situation produces the least negative effect on the caregiver. Furthermore, the promotion of the enhancement of the quality of life and well-being of family caregivers has become imperative, as these individuals frequently become secondary or invisible patients (Pearlin et al., 1990; Schulz et al., 2000).

There has been a broad consensus that dysfunctional thoughts related to caregiving, the frequency, and satisfaction with rewarding activities, social support, social and problem-solving skills, and self-efficacy are important buffering variables. All of these play a relevant role in the appearance of negative effects such as overload and depressive symptomatology (e.g., Ruisoto et al., 2019; Zhong et al., 2020). For this reason, this study has focused on the evaluation of these buffer variables and the main negative consequences related to care that most explanatory theoretical models contemplate (overload, depressive symptomatology, stress, and decreased quality of life) to identify the comprehensive profile of the family caregivers and to direct intervention efforts.

Only recently, some researchers have analyzed the existence of different profiles among family caregivers of people with dementia according to psychosocial and resource variables. Their findings have underscored the importance of kinship groups in each of the profiles and that there are differences in emotional distress among these profiles based on the kinship with the care recipient (Huertas-Domingo et al., 2024). The identification of variables that contribute to explaining caregivers’ distress varies depending on the relationship with the person cared for (Huertas-Domingo et al., 2023). This suggests that focusing on the kinship group is necessary to develop a comprehensive profile of the caregiver.

This study aimed to offer a comprehensive view of the profile of the family caregiver of people with dementia by considering aspects/variables contemplated in theoretical explanatory models of care. These variables represent both the background and current context in which a caregiver operates (variables on socio-demographic characteristics and related to care), as well as the variables that represent the negative repercussions of caregiving, but also those that represent the buffer variables of these. Most studies do not examine all these variables and offer only a fragmented or incomplete analysis. Usually, concerning the profile of a caregiver, only socio-demographic variables are considered such as age, gender, kinship, and educational level among others. Other variables, such as the buffer variables (e.g., dysfunctional thoughts) or the representative of the repercussions of the care situation (e.g., stress) are not contemplated. By studying all these factors, a better understanding of a caregiver's well-being and needs can be achieved, establishing a more integrated profile. This information can then be used to design and implement actions and useful interventions. The objective of this study was to offer a comprehensive profile of the family caregivers of people with dementia that considers the aforementioned variables.

## Methods

### Design, procedure, and participants

To carry out this study, a cross-sectional design was used. Caregivers were evaluated for different variables studied at a single time point.

Participants were recruited through eight day-centers and the municipal telecare service of the city of the study (Salamanca, Spain). The sample consisted of caregivers who wanted to participate in the study, provided their contact information so that the main researcher could contact them, and met the inclusion criteria. The recruitment process was conducted from January to April 2019. The study was approved by the Bioethics Committee of the University of Salamanca (Registration Number 454). Written informed consent was obtained from all participants, with confidentiality being explicitly guaranteed. There were no missing data.

The study included 40 family caregivers of people with dementia. The inclusion criteria were as follows: being the main family caregiver of a person with dementia; being either a spouse or offspring of the dementia sufferer; having never participated in a similar study; and the family member with dementia resided at home (non-institutionalized). In contrast, the exclusion criteria were as follows: the individual in question was not a caregiver, was a secondary (non-primary) or professional (non-relative) caregiver; the individual had a kinship relationship other than that of a spouse or offspring (e.g., daughter-in-law); the person in need of care did not have dementia or he/she was institutionalized; and the caregiver was currently participating in another similar study.

### Instruments

The information questionnaire was comprised of the following items: the socio-demographic characteristics of caregivers (gender, age, marital status, educational level, employment status, and the reasons for assuming the role of the caregiver) and their relatives with dementia (gender and age); and variables related to care (relation to the person with dementia, number of dependent people they were caring for, time as a caregiver (years), and the number of days per week and the number of hours per day dedicated to caregiving). The following is a description of the assessment instruments used to evaluate the buffer variables of the consequences of care and the variables of consequences of care (Table S1 of the Supplementary information).

#### Buffer variables of the consequences of care

- Spanish version of the short form of Social Problem-Solving Inventory-Revised (SPSI-R-25) (D'Zurilla et al., 1999). This 25-item self-applied survey assesses problem-solving skills through five subscales that measure five dimensions of social problem-solving (D'Zurilla et al., 1999; Nezu et al., 2014): positive problem orientation (PPO), negative problem orientation (NPO), rational problem solving (RPS), impulsive/careless style (ICS), and avoidant style (AS). It has a Likert-type response format with five response options (from 0 = *not at all true* to 4 = *extremely true*). Higher scores on “PPO” and “RPS” and lower scores on “NPO”, “ICS”, and “AS” indicate good social problem-solving skills; just as, based on the total score, higher scores correspond to better resolution of social problems (Cronbach’s α = 0.86).

- Adaptation of the Leisure Time Satisfaction survey (LTS) (Losada et al., 2015; Stevens et al., 2004). This survey consists of six items related to different leisure activities and assesses the frequency of doing these activities and the degree of satisfaction. The response format again is a Likert-type scale with three response options (0 = *not at all*, 1 = *a little*, 2 = *a lot for the frequency of performance*; and 0 = *not at all satisfied*, 1 = *a little satisfied*, 2 = *very satisfied* for the satisfaction). Higher scores correspond to performing leisure activities more frequently and with greater satisfaction (Cronbach’s α = 0.36 for frequency and Cronbach’s α = 0.71 for satisfaction).

- Spanish version of the Revised Scale for Caregiving Self-Efficacy (RSCSE) (Márquez-González et al., 2009; Steffen et al., 2002). This survey assesses how the caregiver perceives their ability to perform different care activities. It consists of 15 items, divided into three subscales with five items each that evaluate three dimensions of self-efficacy: self-care and obtaining respite, response to disruptive behaviors, and control of bothersome thoughts. High scores indicate high self-efficacy (Cronbach’s α = 0.82, 0.82, and 0.76, respectively).

-*Escala de Habilidades Sociales* (EHS) (Gismero, 2000). This assesses assertive behavior and social skills. It is made up of 33 items, with a Likert-type response format of four response options (from 1 = *I do not identify myself at all; most of the time it does not happen to me or I would not do it*, to 4 = *I strongly agree and I would feel or would act like this in most cases*). The scale has six subscales/factors: self-expression in social situations, defending one’s rights as a consumer, expression of anger or disagreement, saying no and cutting off interactions, making requests, and initiating positive interactions with the opposite sex. The higher the global score, the greater the social skills and assertiveness in different contexts (Cronbach’s α = 0.81).

- Adaptation of the Psychosocial Support Questionnaire (PSQ) (Izal et al., 2003; Reig et al., 1991). This questionnaire evaluates the nature of social networks according to the subjective perception of the subject. It consists of six items with a Likert-type response format with four options (from 0 = *never* to 3 = *always*). The higher the score, the greater the perception of psychosocial support (Cronbach’s α = 0.74).

- *Cuestionario de Pensamientos Disfuncionales* (CPD) (Losada et al., 2006). It evaluates the thoughts, beliefs, values, and attitudes of the caregivers of dependent elderly people that can hamper suitable coping mechanisms when caregiving. In this case, coping has been divided into two factors: surrender-isolation and emotional self-demand-responsibility. This questionnaire consists of 16 items with a Likert-type response format with five response options (from 0 = *totally disagree* to 4 = *totally agree*). A higher score implies a greater presence of obstacles that hamper adequate coping mechanisms (Cronbach’s α = 0.89).

#### Variables of consequences of care

- Spanish version of the Caregiver Burden Interview (CBI) (Martín et al., 1996; Zarit et al., 1980). This instrument measures the degree to which caregivers perceive that their responsibilities have adverse effects on their health, personal and social life, finances, and emotional well-being. It consists of 22 items with a Likert-type response format with five response options (from 1 = *never* to 5 = *almost always*). A higher score corresponds to a higher level of overload (Cronbach’s α = 0.84).

- Center for Epidemiologic Studies-Depression Scale (CES-D) (Radloff, 1977; Vázquez et al., 2007). This evaluates depressive symptomatology in the general population. It consists of 20 items with a four-option Likert-type response format (from 0 = *rarely or never up* to 3 = *most or all the time*), which assesses whether the person has manifested different symptoms during the previous week. A higher score corresponds to greater depressive symptomatology (Cronbach’s α = 0.90).

- Perceived Stress Scale (PSS) (Cohen et al., 1983; Remor, 2006). The evaluates the level of perceived stress by the caregiver during the last month. It has 14 items with a five-option Likert-type response format (from 0 = *never* to 4 = *very often*). The higher the direct score, the higher the level of stress perceived by the caregiver (Cronbach’s α = 0.88).

- World Health Organization Quality of Life Assessment–AGE (WHOQOL-AGE) (Caballero et al., 2013). This survey has 13 items with a five-option Likert-type response format. The response format is a combination of unipolar (they range from the absence of an attribute to its presence, e.g., “none” to “completely”) and bipolar (they utilize both positively and negatively worded responses on a single item, e.g., “very bad” to “very good”). A higher score corresponds to a better quality of life (Cronbach’s α = 0.85).

### Data analysis

A description was made of the socio-demographic characteristics of the family caregivers, and the variables related to care. Means and standard deviations were obtained for quantitative variables and frequencies and percentages for categorical variables.

Chi-square tests and *t*-tests were performed to check whether there were differences based on the degree of kinship in the socio-demographic and care-related variables. Two-factor ANOVAs (kinship and gender) were performed to check if there were differences in the outcome variables. The assumptions of normality and homoscedasticity were checked prior to analysis.

To perform the analyses, the statistical program IBM SPSS Statistics for Windows (version 22.0) (IBM Corp., 2013) was used with a significance level of α = 0.05.

Finally, the essay question (“What are the reasons for taking on the role of caregiver?”) was analyzed through qualitative methodology using inductive thematic analysis (Andréu, 2001; Attride-Stirling, 2001; Braun & Clarke, 2006). This process can be summarized as follows. The most important ideas/categories expressed by the caregivers were identified in the first phase of the qualitative analysis (categorization). Three main categories of analysis and three subcategories were finally established (see Table S2 of the Supplementary information). The coding was performed in the second phase of the analysis. It was conducted by the authors to ensure the credibility of the process. To test the dependability of the category system, a description of them was established to perform the coding. Finally, confirmability was provided for the results through literal fragments of what was indicated by caregivers.

## Results

### Profile of the caregivers in the socio-demographic and care-related characteristics

In the sample, no significant differences were found in relation to kinship (45% spouses, 55% offspring), but they were found in terms of gender (62.5% women, 37.5% men).

When differences based on kinship were considered in the socio-demographic and care-related variables, no significant differences were found in gender, the number of family members cared for, the length of time they have been caring for, the type of assistance available (formal or informal), the relationship with the family member before and since the care situation. Caregivers typically care for a single person, with an average duration of care of approximately 5 years. The majority of caregivers received formal support. The relationship with the relative before the care situation had been normal or one of intimacy and affection, and it remained the same (see Table 1).

**Table 1:** Socio-demographic and care-related characteristics of the sample of caregivers

Variable	Total (*N* = 40) *n* (%)	Spouse (*N* = 18), *n* (%)	Offspring (*N* = 22), *n* (%)
Caregiver characteristics			
Gender			
Female	25 (62.5)	10 (55.6)	15 (68.2)
Male	15 (37.5)	8 (44.4)	7 (31.8)
Age (years), M (SD), min–max	63.79 (12.16), 39–85	74.4 (6.8), 55–85	55.59 (8.4), 39–69
Marital status			
Single	9 (22.5)	–	9 (40.9)
Married	28 (70)	18 (100)	10 (45.5)
Widowed or divorced	3 (7.5)	–	3 (13.6)
Educational level			
No studies or primary school	12 (30)	9 (50)	3 (13.6)
High school	17 (42.5)	3 (16.7)	14 (63.6)
University	11 (27.5)	6 (33.3)	5 (22.8)
Employment situation			
Active	13 (32.5)	1 (5.6)	12 (54.5)
Not active (unemployed, retired, or other)	27 (67.5)	17 (94.4)	10 (45.5)
Number of persons being cared for			
One	30 (75)	15 (83.3)	15 (68.2)
More than one	10 (25)	3 (16.7)	7 (31.8)
Time as a caregiver (years), M (SD), min–max	5.08 (4.74), 0.8–29	5.47 (3.15), 1–12	4.76 (5.78), 0.8–29
Hours/day as a caregiver, M (SD), min–max	17.05 (7.48), 2–24	20.39 (5.34), 8–24	14.19 (1.97), 2–24
Assistance available			
Formal			
Day center	33 (82.5)	16 (88.9)	17 (77.3)
Home help	18 (45)	8 (44.4)	10 (45.5)
Telealarm or teleassistance	14 (35)	5 (27.8)	9 (40.9)
Informal			
Help from relatives	15 (37.5)	5 (27.8)	10 (45.5)
Help from friends, neighbors, and volunteers	4 (10)	4 (22.2)	–
Help from other people	3 (7.5)	2 (11.1)	1 (4.5)
Time contemplating placing the person in a care home			
Never	20 (50)	13 (72.2)	7 (31.8)
Sometimes or quite often	20 (50)	5 (27.8)	15 (68.2)
Relationship with the family member before caregiving			
Problematic, with conflicts	–	–	–
Quite distant and cold	–	–	–
Normal, under link family that unites us	18 (45)	6 (33.3)	12 (54.5)
Of great intimacy and affection	22 (55)	12 (66.7)	10 (45.5)
Other	–	–	–
Relationship with the family member since becoming their carer			
It has gotten worse	12 (30)	4 (22.2)	8 (36.4)
It has remained more or less the same	21 (52.5)	10 (55.6)	11 (50)
It has improved	7 (17.5)	4 (22.2)	3 (13.6)
Characteristics of family care			
Family member’s gender			
Female	18 (45)	7 (38.9)	11 (61.1)
Male	22 (55)	11 (50)	11 (50)
Family member's age (years), M (SD), min–max	82.62 (7.55), 59–96	77.67 (7.18), 59–87	86.68 (5.09), 75–96

*M* mean *SD* standard deviation, *Min* minimum, *max *maximum

Significant differences were found based on kinship in educational level (*χ*2(2, *N* = 40) = 9.91, *p* = 0.007), employment status (*χ*2(1, *N* = 40) = 10.83, *p* = 0.001), hours per day dedicated to care time (*F*(1.38) = 5.76, *p* = 0.022), and contemplating placing the person in a care home (*χ*2(1, *N* = 40) = 6.47, *p* = 0.011). Spouse caregivers generally had no formal education or had primary education only (Res.tip.corr. = 2.5), were not employed (Res.tip.corr. = 3.3), dedicated an average of 20 h a day to care, and did not consider placing the person in a care home (Res.tip.corr. = 2.5). The offspring caregivers had mid-level studies (Res.tip.corr. = 3.0), were active (Res.tip.corr. = 3.3), spent an average of 14 h a day caring, and sometimes or frequently considered placing the person in a care home (Res.tip.corr. = 2.5).

The following reasons are why participants assumed the role of main family caregiver: (1) owing to an explicit demand; (2) through a group or family decision; and (3) the person decides to take on the role themselves because of reciprocity, gratitude, or as a moral duty or obligation. The frequencies of these reasons reported by caregivers in our study can be seen in Table S2 of the Supplementary information. Spouses assumed caregiving more out of gratitude, moral duty, or obligation in comparison to offspring; while offspring did it to a greater extent out of an explicit demand, though a group or family decision, and reciprocity.

### Profile of the caregivers in the buffer variables and variables of consequences of care

When differences in these variables were analyzed, no kinship-by-gender interaction was significant. Significant differences were obtained based on kinship in self-efficacy for the response to disruptive behaviors, perceived social support, and stress (see Table 2). Spouses presented significantly lower self-efficacy scores for the response to disruptive behaviors. On the contrary, offspring presented significantly lower scores in perceived social support and higher scores in stress. Regarding gender, males had significantly higher scores on dysfunctional thoughts related to caregiving.

**Table 2:** Descriptives of the scores in the outcome variables (buffer variables and variables of consequences of care)

Variable	Total (*n* = 40) M (SD)	Spouses (*n* = 18) M (SD)	Offspring (*n* = 22) M (SD)	*F* (1, 38)	η^2^_p_	Males (*n* = 15) M (SD)	Females (*n* = 25) M (SD)	*F* (1, 38)	η^2^_p_
Buffer variables									
Problem-solving	11.80 (2.11)	11.69 (1.37)	11.88 (2.60)	0.029	0.001	11.89 (1.13)	11.74 (2.55)	0.077	0.002
Frequency rewarding activities	5.33 (1.76)	5.44 (1.34)	5.23 (2.07)	0.517	0.014	5.20 (1.52)	5.40 (1.92)	0.123	0.003
Satisfaction rewarding activities	6.05 (2.94)	6.39 (2.81)	5.77 (3.09)	0.114	0.003	7.13 (2.67)	5.40 (2.96)	3.0	0.077
Self-efficacy self-care and getting respite	181.00 (116.62)	200.00 (103.81)	165.45 (126.37)	0.276	0.008	191.33 (92.34)	174.80 (130.45)	0.070	0.002
Self-efficacy response to disruptive behaviors	209.75 (127.35)	157.22 (126.16)	252.73 (113.73)	5.714*	0.137	218.67 (126.54)	204.40 (130.13)	0.484	0.013
Self-efficacy control of dysfunctional thoughts	260.25 (109.30)	267.22 (128.83)	254.55 (93.13)	0.071	0.002	262.67 (124.35)	258.80 (101.91)	0.002	0.000
Assertiveness	79.95 (13.69)	77.06 (12.43)	82.32 (14.50)	1.930	0.051	77.40 (12.92)	81.48 (14.17)	0.667	0.018
Social support	9.47 (3.58)	11.11 (3.14)	8.14 (3.41)	6.532*	0.154	9.27 (3.45)	9.60 (3.72)	0.520	0.014
Dysfunctional thoughts	31.05 (13.70)	35.50 (12.67)	27.41 (13.70)	3.150	0.080	39.47 (14.02)	26.00 (10.93)	10.32 **	0.223
Variables of the consequences of care									
Overload	62.53 (11.88)	60.11 (9.28)	64.50 (13.57)	1.459	0.039	63.87 (11.70)	61.72 (12.15)	0.485	0.013
Depressive symptoms	23.20 (10.89)	21.61 (9.73)	24.50 (11.82)	0.291	0.008	23.80 (9.73)	22.84 (11.71)	0.182	0.005
Stress	26.60 (9.37)	22.06 (6.91)	30.32 (9.61)	7.623**	0.175	25.73 (8.02)	27.12 (10.22)	0.007	0.000
Quality of life	65.78 (11.63)	69.43 (8.60)	62.80 (13.06)	2.66	0.069	66.40 (10.66)	65.41 (12.37)	0.000	0.000

*p < 0.05. **p < 0.01. ***p < 0.001. *M* mean, *SD* standard deviation

## Discussion

This study aimed to offer a comprehensive view of the profile of the family caregivers of people with dementia. Variables, considered in the theoretical explanatory models of care, were evaluated and represented the background and context in which the caregiver operates, and variables that represent both the negative repercussions of the care situation and those that buffer them. Knowing the comprehensive profile of the caregiver will allow us to design and carry out interventions that are truly adapted to their needs.

Of interest is the fact that the sample does not present significant differences in relation to kinship but does in terms of gender. A similar number of spouses and offspring caregivers participated, but there were more female than male caregivers. That women act as caregivers more often than men is a recurrent observation in the literature (e.g., del-Pino-Casado et al., 2011; IMSERSO, 2015). The unequal distribution of opportunities and responsibilities may be a contributing factor to women being more frequently assigned the role of caregiver (Swinkels et al., 2019).

Caregivers in the sample are distributed equally between spouses and offspring. There are more women than men, who usually care for a single person, having cared for him/her for an average of 5 years and having more formal than informal support. It is possible that the large number of years of caring helps to understand why these caregivers request help despite having formal support. Research indicates that care is often family-based and that the support provided through formal services is still often limited (IMSERSO, 2016). Likewise, the perception of having informal help was also scarce, whether it was from family, friends, neighbors, volunteers, or other people. This fact underscores the necessity for caregivers to receive social support, particularly given the isolation and/or reduction in social relationships that they frequently encounter (Wyatt-Brown, 2015). Moreover, in our study, the relationship with the relative before the care situation has been normal or one of intimacy and affection, and it remains the same. Although the care situation can also contribute to changes in the caregiver-patient relationship (Quinn et al., 2012), this has not been confirmed in this study.

Kinship seems to be the variable that explains the differences in the socio-demographic profile of caregivers and in variables related to care. S*pousal caregivers* had no studies or were primary caregivers, they were not active, spent an average of 20 h a day on care and did not plan to admit the person to a care home*.* In contrast, *offspring caregivers* had secondary education, were employed, spent an average of 14 h a day caring for, and sometimes or frequently thought of placing the person in a care home. The findings of previous research (e.g., IMSERSO, 2015) indicate that caregivers dedicate a considerable number of hours to the care of family members. This finding aligns with the results reported by Jacobs et al. (2016), which demonstrate that, in comparison to other types of caregivers, spouses demonstrate a significantly higher level of dedication to caring for their partner than offspring.

The negative consequences that caregiving can have on the carer can often lead the person to think about placing the family member in a nursing home. This phenomenon has been observed in this study’s offspring sample, but not in spousal caregivers. Spouses may have fewer work responsibilities, allowing them to devote more time and effort to caregiving. Offspring, on the other hand, may feel overburdened by the double or triple shift of combining work, family care, and even care for their own offspring, which may increase the pressure to institutionalize the family member (Li et al., 2021). Also, spouses tend to be older and maybe more aligned in age with the person requiring care. This similarity in age may influence perceptions of moral duty and obligation towards long-term care at home. On the other hand, offspring tend to have greater personal and social demands, which may reduce the time available for caregiving, increasing stress and the perception that assisted living is a necessary option. Spouses may receive greater emotional and logistical support from close networks, which strengthens their ability to maintain care at home. However, offspring, who are often younger, may perceive less support or rely more on formal services. This perceived lack of support also contributes to increased stress and to considering an institution as an alternative to care (Cohen et al., 2019).

The classification of the responses given on the reasons why people assume the role of the caregiver also corresponds with those found in the literature (IMSERSO, 2010; Izal et al., 2003). Besides, our research clarifies the reasons for caring associated with the kinship group: Spouses assumed care to a greater extent out of gratitude or out of a moral duty or obligation compared to the offspring; while the offspring did so to a greater extent by an explicit demand, though a group or family decision, and reciprocity. Familism is a cultural value that has been shown to be important for understanding the dementia caregiving process. It refers to the importance and centrality of the family in an individual’s life, where family bonds and the collective well-being of the family take precedence over individual interests. It is a value that emphasizes unity, solidarity, and mutual support within the family, and it can be seen as a cultural trait that influences people's decisions and behaviors. It is associated with the idea that family members should care for and support one another, even if it requires sacrificing certain personal interests. This may include shared responsibility for the care of the elderly, emotional support, financial assistance, and collective decision-making (Losada et al., 2020).

The values obtained for the buffer variables and the variables of consequences of care coincide with those found in numerous other studies that detect overload, depressive symptomatology, stress, and a lower quality of life in the case of caregivers (e.g., Wyatt-Brown, 2015). This also includes studies that have found a decrease in caregivers’ social and problem-solving skills, social support, rewarding activities, and self-efficacy (Ruisoto et al., 2019; Zhong et al., 2020). Otherwise, the kinship group helps to differentiate the situation and the concrete needs of caregivers in some of these variables. Spouses' caregivers present significantly lower self-efficacy for responding to disruptive behaviors of their couple meanwhile offspring caregivers present significantly lower perceived social support and higher stress. On the other hand, regarding gender, men caregivers present higher dysfunctional thoughts related to care than the women caregivers.

In a similar line, recently Huertas-Domingo et al. (2023), after assessing several kinship groups in socio-demographic variables, familism (family obligations), dysfunctional thoughts, social desirability, frequency and discomfort associated with problematic behaviors, guilt, and depressive symptoms, found that the variables that contribute to explaining caregivers’ distress vary depending on the relationship with the person cared for. This observation underscores the necessity for individualized interventions depending on the kinship group.

Effective interventions for caregivers must contemplate a holistic profile to discern between the most efficient contents, characteristics, and methodology. The literature has shown that psychoeducational interventions are among the most effective (e.g., Bustillo et al., 2018), due to the active role played by the caregiver. Through practical training involving exercises and homework, caregivers can learn how to take care of themselves. It has been observed that various variables are affected in caregivers, and their specific training can lead to mitigate or reduce the negative consequences of care or promote positive consequences, given the relationship that different studies have found between these buffer variables and those of consequences of care (e.g., Ruisoto et al., 2019).

As posited by Tatangelo et al. (2018), interventions with a small number of sessions that encourage caregivers to participate and to continue with the intervention, group-based as they indirectly work on relationships and social support -especially for offspring caregivers- and which have an appropriate selection of content -focusing on strategies for responding with self-efficacy spousal caregivers’ disruptive partner behaviors; focusing on strategies for reducing stress for offspring caregivers; reinforced work to reduce dysfunctional thoughts in men caregivers-, could be the most suitable.

Consequently, the design and implementation of group psychoeducational interventions that take place outside the home and whose content focuses on stress, dysfunctional thoughts, problem-solving skills, and managing difficult family behaviors, social skills, and rewarding activities, seems to be the most effective approach that is adapted to the specific needs of caregivers, considering the kinship with the familiar cared for.

It is acknowledged that this study presents certain limitations. The reduced sample size, attributed to participant non-participation (Jiménez et al., 2022), and the geographical specificity of the sample to the study city may affect the generalizability of the results. However, the socio-demographic profile of the sample is consistent with previously reported data. Future research should prioritize recruiting a larger, more geographically diverse sample to enhance the external validity and provide a more comprehensive understanding.

## Conclusions

Assessing the socio-demographic and care-related characteristics together with buffer variables and negative consequences of care, posed by theoretical models, is important to determine a comprehensive profile of the family caregiver. This allows us to identify the specific needs of caregivers and important constructs that interventions addressed to the caregiver can address. The kinship group with the cared-for relative appears as a relevant variable to understand the reasons for caring, the context and expectations of transition in care, and the emotional distress and personal difficulties that continuous care entails.

This study may offer valuable contributions to scientific research by helping clarify and present a more complete and detailed profile of primary family caregivers of individuals with dementia. Such a profile can assist professionals and researchers in designing and implementing programs tailored to meet caregivers' specific needs based on their kinship group, ultimately improving their circumstances by addressing these variables directly. The study also suggests key guidelines to support this objective.

## Supplementary Information

Additional file 1. Supplementary Table S1 Outcome variables assessed and the instruments used. Supplementary Table S2 Examples of the reasons that led the participants to assume the role of caregiver.

## Data Availability

The datasets generated and/or analyzed in the current study are available from the corresponding author upon reasonable request.

## Abbreviations

AS: Avoidant Style
CBI: Caregiver Burden Interview
CES-D: Center for Epidemiologic Studies-Depression Scale
CPD: Cuestionario de Pensamientos Disfuncionales
EHS: Escala de Habilidades Sociales
HIV: Human Immunodeficiency Virus
ICS: Impulsive/Careless Style
IMSERSO: *Instituto de Mayores y Servicios Sociales*
LTS: Leisure Time Satisfaction
NPO: Negative Problem Orientation
PPO: Positive Problem Orientation
PSQ: Psychosocial Support Questionnaire
PSS: Perceived Stress Scale
RPS: Rational Problem Solving
RSCSE: Revised Scale for Caregiving Self-Efficacy
SPSI-R-25: Short form of Social Problem-Solving Inventory-Revised
WHO: The World Health Organization
WHOQOL-AGE: World Health Organization Quality of Life Assessment – AGE
