# Supplementary material for: Towards a comprehensive psychosocial profile of the family caregiver of people with dementia
Source: Psicol Reflex Crit. 2025 Mar 20;38:6. doi: 10.1186/s41155-025-00342-5 (PMC11925825; doi:10.1186/s41155-025-00342-5)
Supplement: Supplementary file 1 — Additional file 1. Supplementary Table S1 Outcome variables assessed and the instruments used. Supplementary Table S2 Examples of the reasons that led the participants to assume the role of caregiver. [file 41155_2025_342_MOESM1_ESM.docx]

Table S1 Outcome variables assessed and the instruments used

| Variables | Evaluation instruments |
| --- | --- |
| BUFFER VARIABLES | |
| Problem-solving skills | Spanish version of the Short form of Social Problem-Solving Inventory-Revised (SPSI-R-25; D´Zurilla et al., 1999) |
| Frequency and satisfaction with rewarding activities | Adaptation of Leisure Time Satisfaction (LTS; Stevens et al., 2004) by Losada et al. (2015) |
| Self-efficacy | Revised Scale for Caregiving Self-Efficacy (RSCSE; Steffen et al., 2002; Spanish version of Márquez-González et al. (2009)) |
| Assertiveness | *Escala de Habilidades Sociales* (EHS; Gismero, 2000) |
| Social support | Adaptation of the Psychosocial Support Questionnaire (PSQ; Reig et al., 1991) by Izal et al. (2003) |
| Dysfunctional thoughts | *Cuestionario de Pensamientos Disfuncionales* (CPD; Losada et al., 2006) |
| VARIABLES OF CONSEQUENCES OF CARE | |
| Overload | Caregiver Burden Interview (CBI; Zarit et al., 1980; Spanish version by Martín et al., 1996) |
| Depressive symptoms | Center for Epidemiologic Studies-Depression Scale (CES-D; Radloff, 1977; Spanish version by Vázquez et al., 2007) |
| Stress | Perceived Stress Scale (PSS; Cohen et al., 1983; Spanish version by Remor, 2006) |
| Quality of life | World Health Organization Quality of Life Assessment – AGE (WHOQOL-AGE; Caballero et al., 2013) |

Table S2 Examples of the reasons that led the participants to assume the role of caregiver

| Reasons for care | Spouse (N=18),  *n* (%) | Offspring (N=22), *n* (%) | Examples |
| --- | --- | --- | --- |
| 1. By explicit demand | - | 1 (4.5) | "*A cultural issue in my family and that my father asked me especially when he died quickly but not to leave her (her mother) alone*" (Daughter, 58 years). |
| 2. Through a group or family decision | - | 5 (22.7) | “*I am the only relative (son) who lives in (…). My brothers are out (…). The "agreement" was that I would take care of my parents (my father passed away 2 and a half years ago) and so the matter continues*” (Son, 59 years).  “*Because my brothers imposed it on me for being single and living under the same roof*” (Daughter, 47 years).  “*Because I have always been the one who has been the caregiver, both with my father when he was alive, and now with my mother. Also, because my brother decided that he cannot or will not help with the care*” (Daughter, 59 years). |
| 3. Personal choice to become the caregiver |  |  |  |
| Due to reciprocity | - | 3 (13.6) | “*Because they are my parents and I can't do anything else, they have done it for me” (Daughter, 45 years).*  “*Because he is my father, and I owe him everything, and he would have done it for me. I will take care of him as long as I can, all my life*” (Daughter, 58 years). |
| Due to gratitude | 6 (33.3) | 4 (18.2) | “*Because he needed it*” (Daughter, 59 years).  "*Well, love moves me with capital letters and I don't understand it any other way*" (Wife, 80 years). |
| Due to moral duty or obligation | 12 (66.7) | 9 (40.9) | “*Because she was my mother and my obligation as a daughter is to take care of her, without worrying about the responsibility that this entails*” (Daughter, 31 years).  “*Because he is my husband and I feel obligated*” (Wife, 74 years).  “*Because of my obligation as a husband*” (Husband, 72 years). |
